# Major Haemorrhage Following a Transjugular Liver Biopsy: A Case Report and a Discussion of Complications and Learning Points

**DOI:** 10.7759/cureus.31533

**Published:** 2022-11-15

**Authors:** Ertan Teodorescu-Arghezi, Damian Mullan

**Affiliations:** 1 Radiology and Interventional Radiology, The Christie National Health Service (NHS) Foundation Trust, Manchester, GBR

**Keywords:** interventional radiology, patient consent, major complications, haemorrhage, transjugular liver biopsy

## Abstract

Liver biopsy can be performed percutaneously, or via a transjugular approach. Transjugular liver biopsy (TJLB) is usually used in patients who are suffering from severe coagulation disorders (prolonged prothrombin time or low platelets), ascites, severe obesity, or failure of a previous non-targeted percutaneous liver biopsy. In TJLB, the biopsy needle is inserted into the liver parenchyma via the hepatic vein, avoiding transgression of the hepatic capsule and peritoneum. Unlike a percutaneous biopsy, a transjugular approach reduces the risk of bleeding as any bleeding from the biopsy site should be returned into the venous system. It is a safe, well-tolerated procedure, with a major complication rate of less than 0.6%.

This case report describes the rare occurrence of a severe intraperitoneal haemorrhage post-TJLB, and describes and discusses the technique, complication profile, and learning points from this complication.

## Introduction

Transjugular liver biopsy (TJLB)

Liver biopsy is used to assess the nature and severity of acute or chronic liver diseases and can be performed percutaneously, or via a transjugular approach. TJLB was described experimentally in 1964 [1], first performed in 1970 [2], and it is used in those who are suffering from severe coagulation disorders (prolonged prothrombin time or low platelets), refractory ascites, severe obesity, failure of previous non-targeted percutaneous liver biopsy, or for whom there are relative contraindications to percutaneous biopsy, such as amyloidosis [3]. In TJLB, the biopsy needle is inserted into the liver parenchyma via the hepatic vein, avoiding transgression of the hepatic capsule and peritoneum. Unlike a percutaneous biopsy, a transjugular approach reduces the risk of bleeding as the capsule of Glisson is not perforated and any bleeding from the biopsy site should be returned into the venous system [4]. It is a safe, well-tolerated procedure with a major complication rate of less than 0.6% [5].

Technique

Prior to any interventional radiology procedure, written, informed and signed consent must be obtained, explaining clearly to the patient the procedure, the intended benefits, and potential risks and complications. The procedure is performed under local anaesthesia, with or without sedo-analgesia. The patient is placed in a supine position on the angiography table, with the neck exposed and the head turned away from the site of the venous access. Ultrasonographic access of the right internal jugular vein is recommended for increased safety and to avoid complications such as puncture of the ipsilateral carotid artery or pneumothorax and assess the patency of the vessel. The right internal jugular vein is the preferred site of puncture as it provides more direct access to the superior vena cava, inferior vena cava, and hepatic veins [6]. The neck skin is prepared using aseptic technique and after infiltration with local anaesthetic, a small skin incision is made. The right internal jugular vein is punctured with a micropuncture venous access kit comprising an 18 to 21-gauge needle, a 0.018-inch guidewire, and a 5French (Fr) sheath. This allows subsequent placement of a 0.035-inch × 180-cm J-tipped wire to the right atrium and subsequently the inferior vena cava. This is exchanged for an Amplatz super-stiff wire (Boston Scientific, Natick, MA), or equivalent. The Amplatz guidewire is a stainless steel wire with a flat-wire coil and PTFE (polytetrafluoroethylene) coating used to facilitate the placement of a catheter during the procedure. A 9Fr x 35cm guide catheter is then placed over the stiff wire, which allows subsequent placement of a 5-7Fr angled multipurpose catheter to access the right or middle hepatic venous system. A Valsalva technique can be employed to dilate the hepatic veins and improve access. The right hepatic vein is preferentially selected as the biopsy point to ensure a deep puncture more distant from the liver capsule. At this point, a balloon occlusion catheter can be used to occlude the vein to obtain wedged hepatic venous pressure (WHVP) and free hepatic venous pressure, but this is not mandatory. Normal pressure is accepted as less than 5 mmHg. If a satisfactory position is confirmed by venography, the 7Fr biopsy catheter guide can be advanced to the planned biopsy site and the needle deployed. Three Tru-cut samples should be obtained per procedure, with two samples from a more peripheral site and one from a slightly more centralised site [6]. After the biopsy, a hepatic venogram can be performed to exclude any extravasation in the peritoneal cavity. The biopsy equipment is then removed, with manual compression at the puncture site to achieve haemostasis.

Contraindications and complications

As with any interventional procedure, TJLB is not without complications and there are some relative contraindications to it. Abnormal coagulation is not a contraindication, but steps should be taken to correct any severe coagulation abnormalities. Refusal of consent is deemed an absolute contraindication. Thrombosis of the right jugular venous system can preclude access or carry a risk of dislodging clot, and access from the left jugular or femoral route are possible but difficult. Hydatid cysts, cholangitis, and hepatic vein thrombosis are contraindications but can vary, dependent on the anatomical location of cysts, thrombus, and biopsy site [3]. In a systematic review of 7469 TJLBs, it has been shown that the total rate of complications was 7.1% [5]. These were divided into minor complications totalling 6.5%, including neck pain, haematoma, bleeding or carotid puncture. A major complication rate of 0.6% includes a large hepatic haematoma, intraperitoneal haemorrhage, and pneumothorax. A mortality rate of 0.1% is quoted, caused by intraperitoneal haemorrhage and ventricular arrythmia. Considering that TJLB is performed in patients with coagulopathy, the same study concludes TJLB is a safe procedure, with a low major complication rate (0.5%) and mortality (0.09%) [5]. Another study also found that TJLB is safe even in patients with severe coagulopathies and multiple biopsies [7].

With these points in consideration, we present a case of complications arising from TJLB, and discuss the incidence and learning points from this.

## Case presentation

A 64-year-old man presented with progressive fatigue and lymphocytosis on a routine medical examination. Blood count showed elevated white blood cells with immunophenotyping compatible with grade 4 follicular lymphoma. Original staging CT scan showed widespread lymphadenopathy, splenomegaly, and a renal mass, which was later diagnosed as renal cell carcinoma and treated with partial nephrectomy. The patient underwent chemotherapy and was treated with six cycles of bendamustine, rituximab, and rituximab maintenance. Due to disease progression, the patient had salvage chemotherapy with partial remission, followed later by a sibling allogenic stem cell transplant. The patient developed graft-versus-host disease (GVHD) for which he was started on methylprednisolone, ruxolitinib, and mycophenolate. 

**Figure 1:**
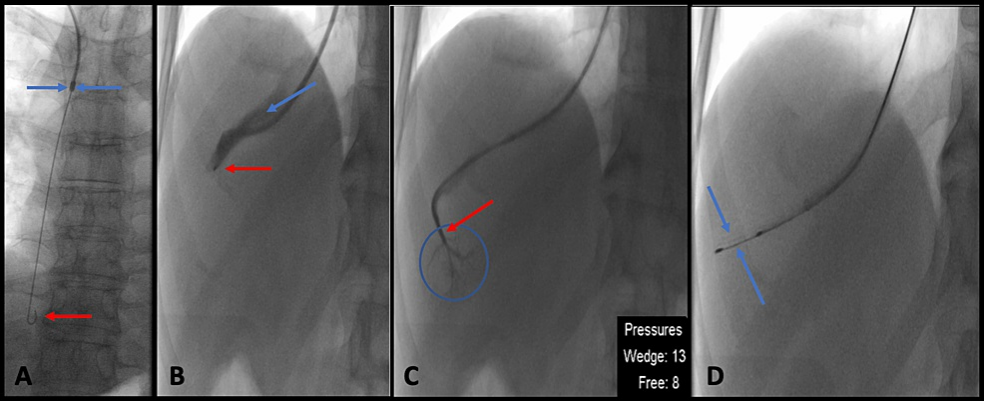
Fluoroscopic images of TJLB procedure (A) 9Fr access catheter passed via right internal jugular vein to superior vena cava (blue arrows), with a stiff 0.035-inch guidewire passed to the right atrium (red arrow ). (B) Venogram of a 5Fr angled multipurpose catheter (red arrow indicating tip of catheter) accessing the right hepatic vein (blue arrow showing vein outlined by iodinated contrast. (C) The catheter (red arrow) can be passed distally into a wedge subsegment (blue circle) and used to estimate free, and wedge hepatic venous pressure. (D) When satisfactory position of the planned biopsy site has been confirmed, the needle (blue arrows) is deployed, and biopsies taken. TJLB: Transjugular Liver Biopsy; Fr: French

The patient developed severe cholestasis, with deranged liver function and clotting markers. A TJLB was planned to assess the nature of the hepatic dysfunction (Figure 1A-1D). Haemoglobin and platelet levels the day prior to the procedure were 6.8g/dl and 20 x 10^9^/L, respectively, and he received two units of packed red blood cells and two units of platelets to bring levels within an acceptable procedural range (8g/dl, and >50 x 10^9^/L in our institution). The patient gave his consent prior to the procedure after the risks and benefits were explained. The procedure was performed under conscious sedation. Hepatic biopsies were obtained and sent for histology. No complications were noted throughout the intervention. 

**Figure 2:**
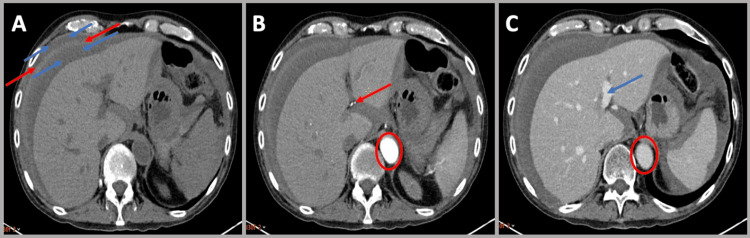
Triple phase CT of the liver following TJLB (A) Unenhanced CT showing hyperdense fluid around the liver (red arrows), surrounded by less prominent hyperdense fluid (blue arrows), indicating intraperitoneal haemorrhage with sedimentation of blood into layers. (B) Arterial phase CT showing arterial phase contrast in the aorta (red circle) and hepatic arteries (red arrow) with no acute extravasation of contrast detectable around the liver. (C) Venous phase CT showing portal venous phase contrast in the aorta (red circle) and hepatic portal veins (blue arrow) with no acute extravasation of contrast detectable around the liver. TJLB: Transjugular Liver Biopsy

**Figure 3:**
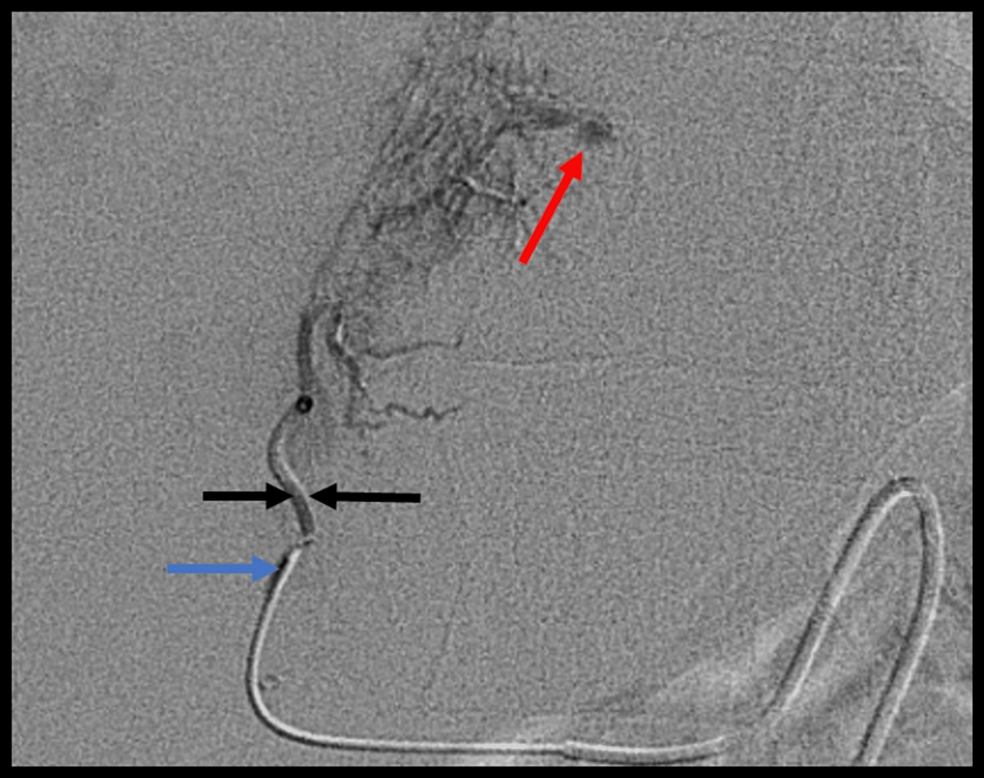
Digitally subtracted angiogram of the liver showing super-selection of a branch of a segment 4 artery (black arrow) with a 2.7Fr microcatheter (blue arrow), and a blush of contrast (red arrow) suggestive of the possible bleeding point Fr: French

Two hours after his return to the ward, the patient became unwell, with hypotension refractory to fluid resuscitation and abdominal tenderness. Haemoglobin levels had dropped to 5.5g/dl, having been 8.9g/dl immediately pre-procedure. Haemorrhage was suspected, and although a clinically uneventful procedure, a capsular breach was suspected post-TJLB. An urgent triple-phase CT scan of the abdomen with iodinated contrast showed a large intraperitoneal haemorrhage, although the exact bleeding point was not clearly visualised (Figure 2A-2C). The patient underwent a hepatic angiography, which showed a triangular arterial blush arising from a small segment 4 right hepatic artery branch, with late venous communication in keeping with the likely source of the bleeding (Figure 3). The small hepatic artery branch was successfully embolized with bland particulate injection, with cessation of the bleeding, and clinical stabilization. Histological analysis of the hepatic biopsies excluded GVHD but showed prominent cholestasis, most likely drug-induced, and associated with iron overload consistent with transfusion siderosis, raising the possibility of haemochromatosis. Ultimately, the patient’s liver function continued to deteriorate, with co-existing sepsis and an episode of melena from lower gastrointestinal haemorrhage. He passed away three weeks post-TJLB. In view of death within one month of an invasive procedure, the case was referred to the coroner for review, with a conclusion that the cause of death was sepsis, acute GVHD, and melena as complications of allogenic stem cell transplantation. The biopsy was felt to have contributed to morbidity post-procedure but was not felt to be the cause of death. 

## Discussion

Patients undergoing TJLB will usually already have comorbidities for which a percutaneous biopsy is not indicated, and can have an overall higher risk of complications, even with a proven safe and well-tolerated procedure. An indication for requiring a TJLB assumes that the general physiological status of the patient is already compromised. Any further insult to the patient as a result of a procedural complication may have a more severe morbidity profile than would be expected in a patient with a normal physiological/clotting profile. TJLB procedures require foreplanning of anatomy and potential anatomical or pathological variance which might impact an individual procedure. 

Major complications are reported in 0.6% of TJLB patients with a reported mortality rate of <0.1% for adults. Major bleeding complications of TJLB are extremely rare with a very low incidence reported in the literature. Mortality may be related to haemorrhage from an extracapsular liver puncture, biopsy perforation of the hepatic artery, or cardiac arrhythmia. Other rare complications can include haemobilia or pseudoaneurysm, or capsular perforation after a wedge injection of contrast [5,8,9].

Although the liver biopsy did not directly cause the patient’s death in this case, studies have shown that major bleeding with a requirement for blood transfusion is linked to increased 30-day mortality [10,11]. Early detection followed by embolization of the bleeding vessel leads to a favourable outcome; however, there are isolated cases where a fatal outcome has been reported [4]. In this case, the patient had fully consented to all risks of TJLB, including haemorrhage. Consent should be obtained according to national guidelines. This should be both verbal, and in written form, with a careful explanation of the benefits and risks involved. Consent must be obtained in sufficient time prior to the procedure so that the patient can process the information and make an informed decision [12,13]. Risks of very severe but rare morbidity and mortality should be discussed irrespective of incidence [14]. If sedo-analgesia is to be given during any procedure, consent must be given well in advance of the procedure to allow prospective retention and avoid the retrospective memory loss associated with some sedation drugs [15].

Despite the low haemorrhagic risk associated with TJLB, low haemoglobin (<8g/dl) should be corrected pre-procedure to a level that is survivable should a severe haemorrhagic complication occur. Low platelet levels (<50 x 10^9^/L) should be corrected to aid the clotting cascade should a severe haemorrhagic complication occur. 

The patient was transfused two units of blood pre-procedure to optimise haemoglobin to a level that would physiologically tolerate a complication of haemorrhage to a survivable threshold. There was intraperitoneal haemorrhage despite pre-procedural platelet transfusion up to a level of 99 x 10^9^/L. A platelet level of over 50 x 10^9^/L is an accepted lower threshold for many procedures. It may be a reassuring number for an operator performing an invasive procedure; however, it only mitigates the risk of bleeding, rather than definitively preventing it. Operators should consent patients for haemorrhagic risks irrespective of blood parameters.

During any radiological procedure, sequential images of the key steps should be saved to act as confirmatory evidence that the procedure has evolved as planned with respect to the technical and anatomical steps of the procedure. This acts as a visual and evidential record of the procedure, should the case require retrospective review, or if a case of complication is escalated to medico-legal experts for any reason [16]. Selected fluoroscopic images had been prospectively saved and formally recorded in this case. A venogram post-biopsy was performed as standard practice to exclude post-biopsy bleeding in this case. However, the real-time cine-loop images of the post-biopsy venogram were not formally recorded or saved to definitively prove a lack of post-biopsy bleeding on a cold retrospective review of this case.

Post-procedure care should mandate regular checks of physiological status, even for procedures such as TJLB with very low documented risks of haemorrhage. In this case, aftercare protocol was adhered to and was sufficient to detect the early clinical signs and symptoms of significant intraperitoneal haemorrhage, allowing timely intervention to embolize the bleeding point. 

## Conclusions

TJLB is a safe and well-tolerated procedure in patients with liver disease and contraindications to percutaneous liver biopsy. Although the incidence of severe haemorrhage following TJLB is very low, it is important to remember that every procedure carries risks. Informed consent, obtained well in advance of the procedure is crucial. Patients need to be explicitly aware of the intended benefits, common and severe risks, and complications, and be consented appropriately, and in advance of the procedure. Consent should be clearly documented, and coagulopathy should be corrected as much as possible. Prospective recording of all key procedural images and stages should be performed as a gold standard to serve as evidential tools of the evolution of the procedure should any complication arise.
